# Synthetic Analogue of Butenolide as an Antifouling Agent

**DOI:** 10.3390/md19090481

**Published:** 2021-08-25

**Authors:** Ho Yin Chiang, Jinping Cheng, Xuan Liu, Chunfeng Ma, Pei-Yuan Qian

**Affiliations:** 1 Department of Ocean Science, Division of Life Science and Hong Kong Branch of the Southern Marine Science and Engineering Guangdong Laboratory (Guangzhou), The Hong Kong University of Science and Technology, Hong Kong, China; hoyinchiang@ust.hk (H.Y.C.); jincheng@ust.hk (J.C.); ocesxliu@ust.hk (X.L.); 2 Faculty of Materials Science and Engineering, South China University of Technology, Guangzhou 510000, China; msmcf@scut.edu.cn; 3 Southern Marine Science and Engineering Guangdong Laboratory (Guangzhou), Nansha 510000, China

**Keywords:** antifouling compounds, structural optimisation, butenolide, larval attachment assay

## Abstract

Butenolide derivatives have the potential to be effective and environmentally friendly antifouling agents. In the present study, a butenolide derivative was structurally modified into Boc-butenolide to increase its melting point and remove its foul smell. The structurally modified Boc-butenolide demonstrated similar antifouling capabilities to butenolide in larval settlement bioassays but with significantly lower toxicity at high concentrations. Release-rate measurements demonstrated that the antifouling compound Boc-butenolide could be released from polycaprolactone-polyurethane (PCL-PU)-based coatings to inhibit the attachment of foulers. The coating matrix was easily degraded in the marine environment. The performance of the Boc-butenolide antifouling coatings was further examined through a marine field test. The coverage of biofouler on the Boc-butenolide coatings was low after 2 months, indicating the antifouling potential of Boc-butenolide.

## 1. Introduction

Since the prohibition of tributyltin in 2008, many studies have attempted to discover novel antifouling compounds from marine natural products [1]. Many bioactive marine natural products have been screened and tested in recent decades, and several reviews on marine natural products and their synthetic analogues as antifouling compounds have been published [2,3,4,5]. Although many potent antifouling compounds have been discovered, those compounds are rarely commercialised. The low supply of antifouling compounds has hindered the development of antifouling paints based on the marine natural products [2,3,6,7]. Two solutions to the problem exist. The first solution is to explore marine natural products from microorganisms, as microorganisms can produce a wide range of bioactive secondary metabolites [8,9,10]. The convenience of bacterial cultivation and the mass production of metabolites in a short period of time have benefits over the extraction of compounds from the microorganism [6,7,10]. The second solution involves structural optimisation using organic synthesis [6,11]. Secondary metabolites extracted from organisms are often complex in structure; thus, they can be difficult to synthesise effectively in large quantities for commercial-scale usage [6,11]. By studying the structure–activity relationship of bioactive compounds isolated from organisms, pharmacophores that are responsible for antifouling abilities can be identified [11]. Optimisation of the compound’s structure is performed with the goal of increasing its potency, decreasing the toxicity of the original compound [12], improving other physical or chemical properties of the compound and simplifying the chemical structure for chemical synthesis.

**Figure 1:**
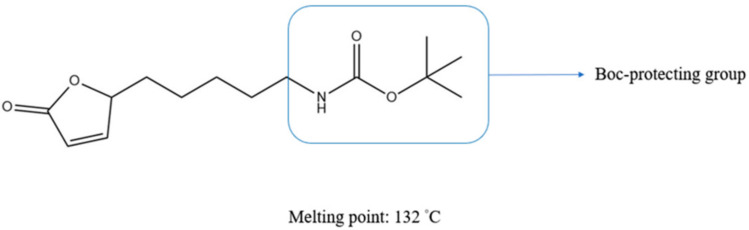
Chemical structure of *tert*-butyl (5-(5-oxo-2,5-dihydrofuran-2yl)pentyl)carbamate (Boc-butenolide).

The antifouling compound 5-octylfuran-2(5H)-one (butenolide) has a melting point at 23 °C, which causes it to change from solid form to liquid at high ambient temperatures. In the present study, a butenolide derivative was modified with a Boc-protecting-group at the terminal of the alkyl side chain, *tert*-butyl (5-(5-oxo-2,5-dihydrofuran-2-yl)pentyl)carbamate (Boc-butenolide) (Figure 1). This modification aims to improve its environmental stability and to remove its foul smell by increasing its melting point from 23 °C to 132 °C. A higher melting temperature above ambient temperature could lead to a more precise control of the proportion of antifoulant added during coating formulation. The modified Boc-butenolide (Figure 1) was further characterized for its melting point, stability and antifouling bioactivity using anti-larval settlement bioassays with larvae of barnacles (*Amphibalanus* *amphitrite*) and tubeworms (*Hydroides elegans*) in the laboratory. The modified Boc-butenolide was further formulated into an antifouling paint using polymer matrix poly(ε-caprolactone) based polyurethane (PCL-PU). Release-rate measurements of Boc-butenolide and a marine field antifouling test were conducted to evaluate the feasibility of using Boc-butenolide as an active ingredient in antifouling coatings.

## 2. Results and Discussion

### 2.1. Stability and Solubility of Boc-Butenolide and Butenolide

**Figure 2:**
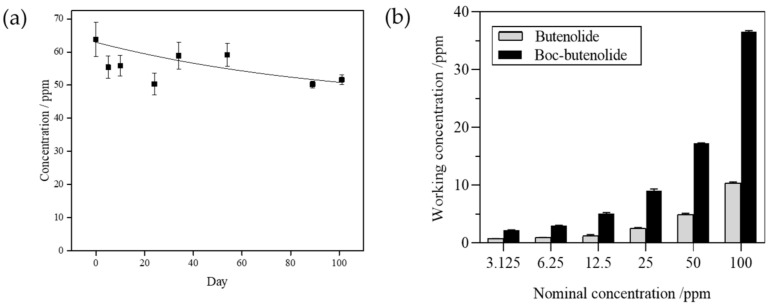
Measured concentrations of Boc-butenolide in ASW for 3 months (**a**), and working concentrations of butenolide and Boc-butenolide (**b**) used in Figures 3 to 6.

Figure 2a illustrates the measured concentrations of Boc-butenolide in ASW for 3 months. The concentration of Boc-butenolide measured at day 0 is around 64 ppm and dropped to approximately 50 ppm after 3 months in ASW. In the following anti-larval settlement bioassay experiments, the same nominal concentrations for each condition were used to compare their bioactivity. To understand the relationship between nominal concentration and soluble or working concentrations of the two compounds, the working concentrations for all nominal concentrations used in the experiments were tested using HPLC. Figure 2b shows that the measured working concentrations of Boc-butenolide are higher than all tested working concentrations for butenolide under all tested nominal concentrations (3.125, 6.25, 12.5, 25, 50, and 100 ppm). The average dissolution rate of Boc-butenolide is approximately 40%, while the average dissolution rate of butenolide is only around 10%.

### 2.2. Antifouling Performance of Boc-Butenolide

**Figure 3:**
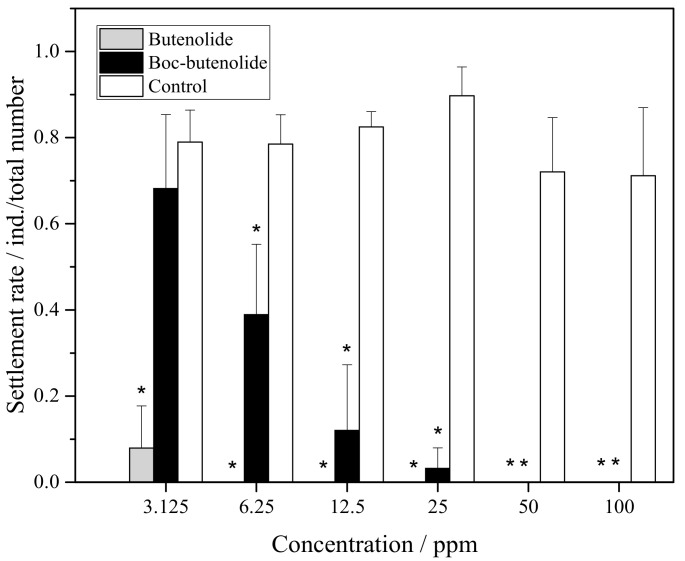
*A. amphitrite* larval settlement rate after Boc-butenolide and butenolide treatments. Asterisks indicate a significant difference from the control with *p* < 0.05.

Figure 3 shows the settlement rate of *A. amphitrite* larvae treated with Boc-butenolide and butenolide. At nominal concentrations of 50 ppm to 100 ppm, both compounds inhibited the settlement of barnacle cyprids. Settlement rates between the two compounds began to differ at 25 ppm. For Boc-butenolide, the cyprids showed some settlement at a rate of approximately 3%. The settlement rate continuously increased, reaching approximately 70% at a concentration of 3.125 ppm. For butenolide, the attachment of cyprids was inhibited between the concentrations of 6.25 ppm and 100 ppm, and approximately only 10% of the larvae settled in the treatment of 3.125 ppm.

**Figure 4:**
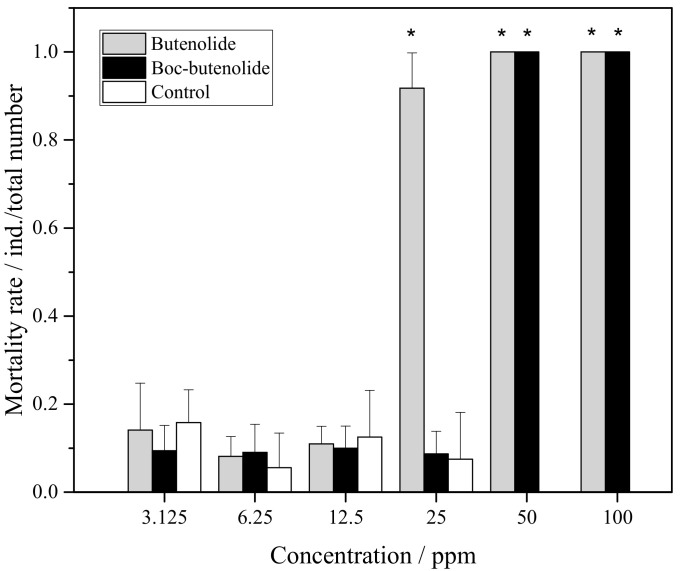
*A. amphitrite* larval mortality rate after Boc-butenolide and butenolide treatments. Asterisks indicate a significant difference from the control with *p* < 0.05.

Figure 4 shows the mortality rate of *A. amphitrite* larvae treated with Boc-butenolide and butenolide. For both compounds, there was no significant difference in toxic effects compared with the control group between the nominal concentrations of 3.125 ppm and 12.5 ppm. A pronounced difference in toxic effects was observed at 25 ppm. A very low toxicity was observed for Boc-butenolide-treated larvae, i.e., the mortality rate was approximately 8%, compared with the obvious toxic effect observed for the butenolide-treated larvae, in which mortality rate was over 90% among the individuals at high concentrations. All cyprids died in both treatments with higher antifoulant concentrations (50 and 100 ppm), indicating the toxic effects of the antifouling compounds at high concentrations.

**Figure 5:**
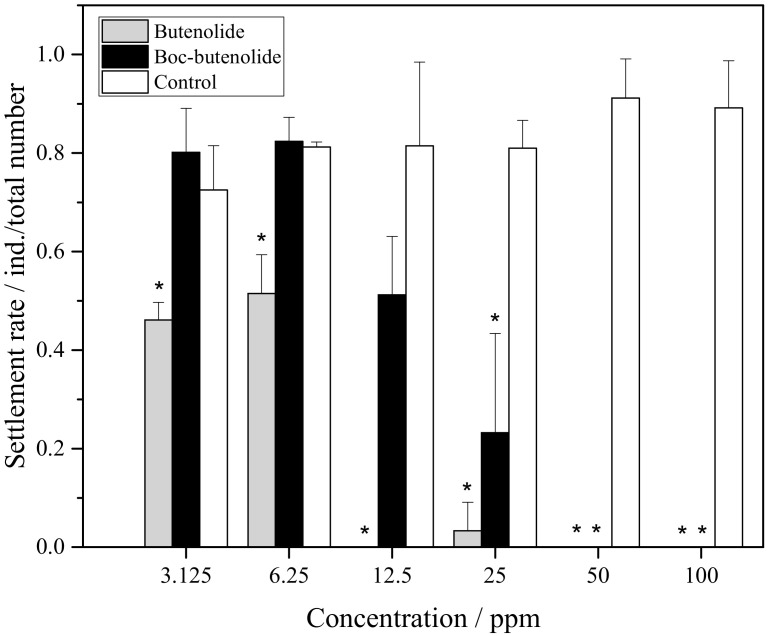
*H. elegans* larval settlement rate after Boc-butenolide and butenolide treatments. Asterisks indicate a significant difference from the control with *p* < 0.05.

The *H. elegans* larval settlement bioassay results were similar to those of the *A. amphitrite* larval settlement bioassay. No settlement of larvae was observed after treatments with Boc-butenolide at 50 and 100 ppm (Figure 5), whereas for butenolide treatments, effective inhibition of larval settlement was found even at a low concentration of 12.5 ppm. For Boc-butenolide treatments, a significant difference from the control group was still observed at 25 ppm, with a settlement rate of approximately 20%. An increase in settlement inhibition was observed from 3.125 ppm to 25 ppm. For butenolide treatments, a significant difference was found between treatment and control groups across all concentrations, with a settlement rate of approximately 50% at low concentrations of 3.125 and 6.25 ppm.

**Figure 6:**
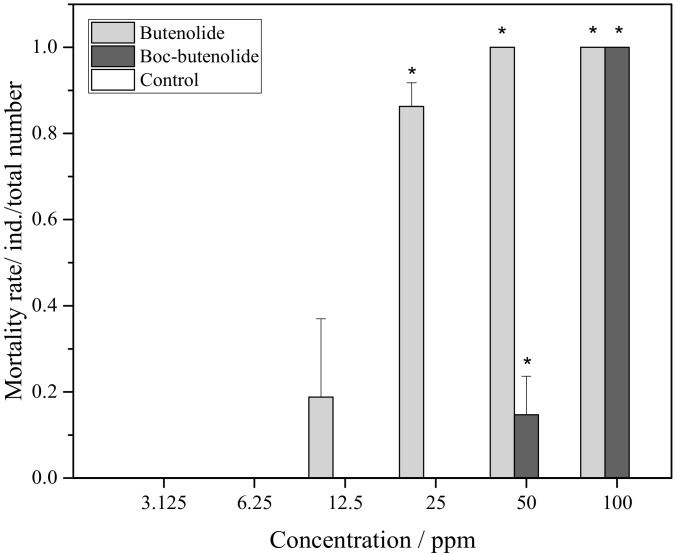
*H. elegans* larval mortality rate after Boc-butenolide and butenolide treatments. Asterisks indicate a significant difference from the control with *p* < 0.05.

In terms of the toxicity of the compounds (Figure 6), only Boc-butenolide treatments at 50 and 100 ppm showed significant toxicity on the *H. elegans* larvae. However, for butenolide treatments, toxic effects started to appear at 12.5 ppm, reaching a 100% mortality rate at concentrations above 50 ppm. Structural differences between Boc-butenolide and butenolide affect the pharmacokinetics of the two compounds, leading to differences in potency and toxicity towards the larvae.

### 2.3. Release Rate of Boc-Butenolide from the Coatings

**Figure 7:**
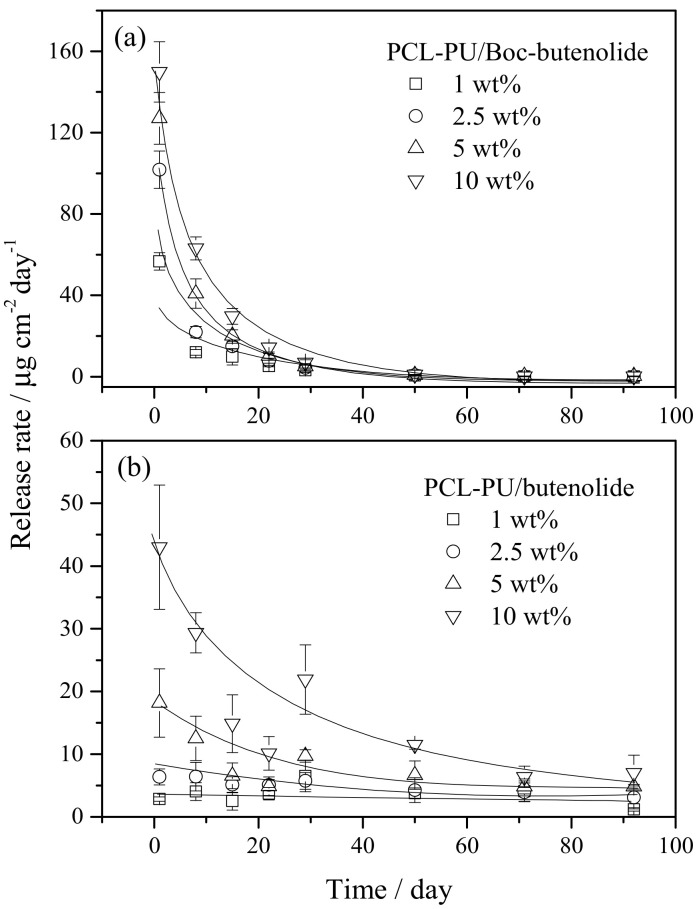
Release-rate measurement of (**a**) Boc-butenolide and (**b**) butenolide with PCL-PU matrix for 90 d.

Figure 7 shows the release rate of different concentrations of Boc-butenolide and butenolide for at least 90 d. Generally, the amount of compounds released from the coatings at a particular time is positively correlated with the initial concentration of the compounds in the coatings. The release rate could be controlled by changing the concentration of antifoulant in the coatings. The initial release rate of Boc-butenolide (150 µg/cm^2^/day) was much higher than that of butenolide (45 µg/cm^2^/day) for 10 wt% samples. A possible explanation is the hydrophilicity difference between Boc-butenolide and butenolide. A Boc-protecting-group was added to the side chain of butenolide. Thus, the Boc-butenolide was more hydrophilic with the added highly electronegative N and O atoms of the Boc group and easier to dissolve in seawater, thereby resulting in a high initial release rate. A huge decrease in the release of antifoulant from the coatings for both Boc-butenolide and butenolide was found after 1 month. Notably, the release of Boc-butenolide from the coatings for all four concentrations was much lower than that of butenolide after 1 month. This finding might be due to the fact that a steady release in the later stage cannot be supported after a huge initial release of Boc-butenolide.

PCL-PU polymer is biodegradable and environmentally friendly, and was applied as the polymer to develop the antifouling coating system in the present work. In the previous study, a PCL-PU/butenolide antifouling coating system was developed and test results showed that the polymer coating could be degraded in the sea [13]. In this experiment, PCL-PU/Boc-butenolide showed a large decrease in release rate when compared to that of PCL-PU/butenolide, which might be due to the compatibility of Boc-butenolide in PCL-PU and the relatively high solubility of Boc-butenolide in seawater. In future studies, more effort should be made in the improvement of the polymer structure so as to optimize the release performance of Boc-butenolide as an antifouling coating.

### 2.4. Field-Test Performance

**Figure 8:**
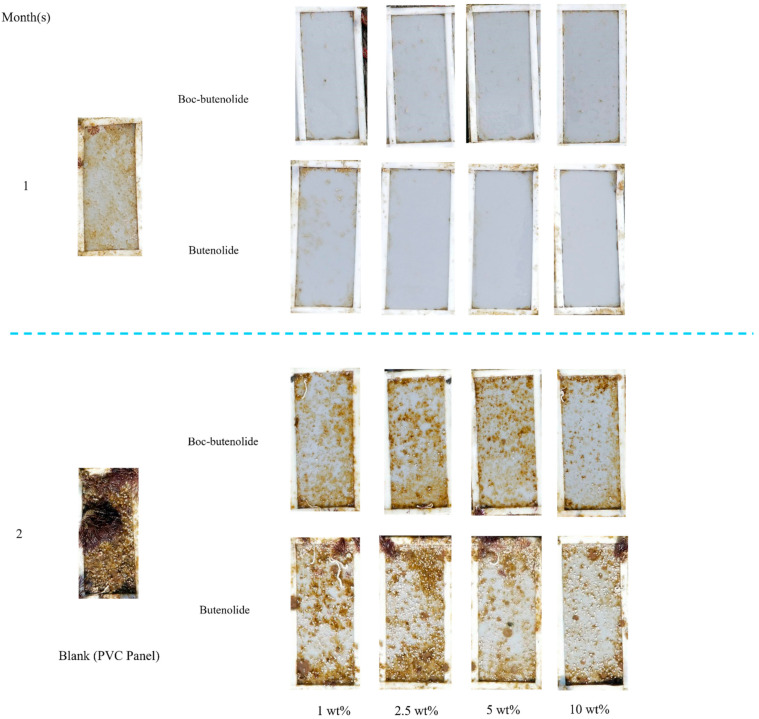
Field test of various concentrations of Boc-butenolide and butenolide with PCL-PU matrix for 2 months. From left to the right, the control, 1, 2.5, 5 and 10 wt% of Boc-butenolide (**top**) or butenolide (**bottom**) are presented. The test was continued for 2 months and retrieved at monthly intervals.

**Figure 9:**
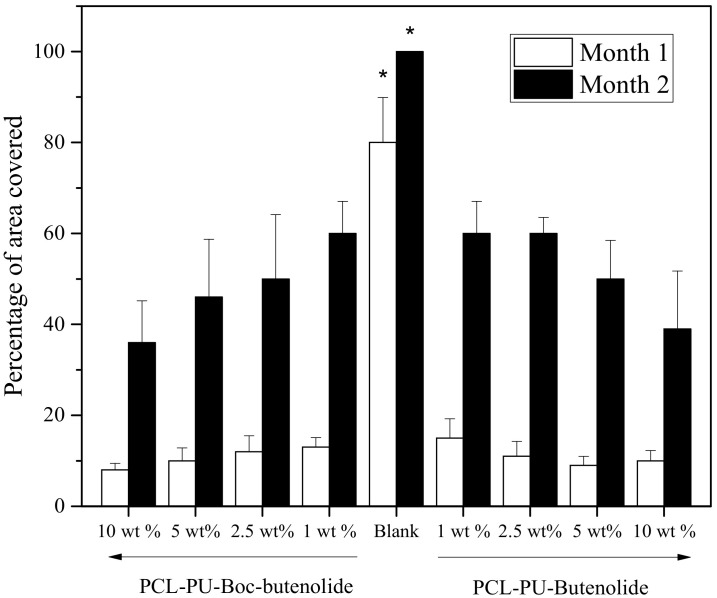
Percentage of area covered with biofoulers for different coatings during a period of 2 months. From left to right, PCL-PU with 10, 5, 2.5 and 1 wt% Boc-butenolide, control and PCL-PU with 1, 2.5, 5 and 10 wt% butenolide are presented. Asterisks indicate a significant difference between the samples within 1 month (*p* < 0.05).

A field test was conducted to evaluate the performance of the coatings in the marine environment. Figure 8 and Figure 9 show the images and the relative coverage of the panels coated with different concentrations of Boc-butenolide or butenolide with PCL-PU as a polymer matrix. After 1 month of exposure, all of the coatings with antifoulants remained almost fouling-free, with less than 10% coverage. Approximately 80% biofilm coverage was observed on the control panels. All of the panels treated with the coatings showed good antifouling performances, indicating the effectiveness of Boc-butenolide and butenolide as antifoulants. This finding is consistent with the larval settlement results, suggesting that the compounds can prevent larval attachment.

In the second month’s results, the coatings with higher concentrations of Boc-butenolide or butenolide showed better antifouling abilities (Figure 9). Performance levels of both Boc-butenolide and butenolide were similar. These results were consistent with the release rate and larval attachment results mentioned above. Although Boc-butenolide was less potent in preventing larval settlement at similar nominal concentrations in the laboratory studies, its huge amount of initial release helped to compensate for its lack of potency, resulting in an antifouling performance similar to that of butenolide in the field test.

The differences in effectiveness and toxicity between Boc-butenolide and butenolide are possibly due to their structural difference. Considering that a Boc group was added to the carbon side chain of butenolide in Boc-butenolide, its structure was large and bulky. Molecular weight and hydrophilicity can affect the pharmacokinetics of the compounds, especially regarding their absorption by organisms [14]. Generally, a large molecule containing electronegative atoms, such as N or O, is more hydrophilic and is difficult to be absorbed or become bioavailable to the organisms. Therefore, Boc-butenolide shows a lower effectiveness when compared with butenolide. The difference in effectiveness was obvious at 25 ppm. At the same time, as Boc-butenolide has a lower absorption or bioavailability, it also shows no or relatively low toxic effects to the treated larvae. The structural modification of butenolide changes the physical and chemical properties of the original compound. For instance, Boc-butenolide is odourless and with a lower toxicity towards the larvae compared with butenolide. Boc-butenolide’s chemical stability is also increased, as the Boc group is stable towards most nucleophiles and bases. These changes in physical and chemical properties can be beneficial when designing effective antifouling coatings and also when considering the environmental impact for actual use.

From the release-rate results, an exponential decrease in Boc-butenolide release into the seawater throughout the test period was observed. This result may be due to the high hydrophilicity of Boc-butenolide, which allows it to dissolve more easily in the seawater and achieve a high release rate. The release rate can be improved through the structural modifications of Boc-butenolide. Changes in physical and chemical properties, such as the melting point and hydrophilicity, can be achieved by structural modification [12]. For instance, the water solubility of Boc-butenolide can be reduced by a slight structural modification. Another method for optimising the controlled release of Boc-butenolide in seawater is by developing a suitable polymer as a binder. For example, the antifouling compound could chemically bind to the polymer chain, e.g., in tributyltin-SPC antifouling coatings [15,16]. The release of biocide would then be controlled by the hydrolysis of the polymer, thereby controlling the release rate of the biocide.

## 3. Materials and Methods

### 3.1. Chemicals and Seawater

All chemical reagents used in this study, unless otherwise specified, were purchased from Sigma Aldrich (St. Louis, MO, USA) and VWR chemicals (Haasrode, Belgium). 5-Octylfuran-2(5H)-one (butenolide) and *tert*-butyl (5-(5-oxo-2,5-dihydrofuran-2-yl)pentyl)carbamate (Boc-butenolide) with a purity of >99% were purchased from ChemPartner (Shanghai, China) and used as received. Acetonitrile and methanol used were of HPLC grade.

Seawater was collected using a pump at the Coastal Marine Laboratory of Hong Kong University of Science and Technology. Filtered seawater (FSW) was obtained by filtering seawater through a 0.22 μm filter membrane from Millipore (Merck KGaA, Darmstadt, Germany). Artificial seawater (ASW) was prepared according to ASTM D114198 standards (2013) [17].

The stock concentrations of butenolide and Boc-butenolide were made by dissolving 100 mg of butenolide or Boc-butenolide in 1 mL of DMSO to make a stock of 100 mg/mL, stored at −20 °C. The butenolide and Boc-butenolide samples used in the larval settlement and mortality experiments were prepared by serial dilution of stock concentrations of butenolide and Boc-butenolide using ASW as the diluent, and the DMSO content in final samples were lower than 0.5‰ *v*/*v*.

### 3.2. Collection of Amphibalanus amphitrite Larvae Sample

Adult *A. amphitrite* colonies were collected from Tso Wo Hang Pier (22°23′32.1′′ N 114°17′18.7′′ E), Hong Kong, from April 2018 to June 2018. The adults were kept in a water tank with running seawater at the Coastal Marine Laboratory (the Hong Kong University of Science and Technology) for no more than a week before experimental use. Adults were induced to hatch under light sources for 1 h; the larvae were obtained using a method described previously by Harder et al. [18]. The nauplii larvae newly released from the adults were reared on diatom *Chaetoceros gracilis* Schütt. The seawater culture medium was replaced daily with fresh FSW and algae. The nauplii reached the competent stage, known as cyprid, after 4 d of incubation at approximately 28 °C.

### 3.3. Collection of Hydroides elegans Larvae Sample

Adult *H. elegans* colonies were collected from a fish farm at Yung Shue O, Hong Kong (22°24′ N, 114°21′ E) from March 2019 to April 2019. The adults were kept in a water tank with running seawater at the Coastal Marine Laboratory of Hong Kong University of Science and Technology for no more than 3 d before experimental use. The larvae were collected according to the methods described by Qian and Pechenik [19]. The tube of the adults was gently cracked with forceps to release the gametes. Oocytes were then mixed with the sperm and transferred into a new container with 500 mL FSW for fertilisation. Larvae were reared on microalga *Isochrysis galbana* (Tahitian strain) after hatching. The seawater culture medium was replaced daily with fresh FSW and algae, and the trochophore-stage larvae reached the competent stage after 5 d of incubation at approximately 25 °C.

### 3.4. Larvae Food and Cultivation

The diet for *A. amphitrite* and *H. elegans* cultivated in this study comprised *C. gracilis* and *Isochrysis galbana*, respectively. In the laboratory, the algae were cultured with Guillard’s f/2 medium. The f/2 medium was prepared by adding designated amounts of NaNO_3_, NaH_2_PO_4_ H_2_O, trace metal and vitamin solutions into autoclaved FSW [20]. Na_2_SiO_3_ 9H2O was added for the cultivation of *C. gracilis*. Algal stocks were then added into the culture medium in a 2 L Erlenmyer flask and subcultured bi-weekly. The cultures were bubbled and illuminated under 14 h/10 h light/dark cycle at 23 °C for incubation.

### 3.5. Settlement Bioassay of A. amphitrite

The test compounds were dissolved with a small amount of DMSO. The test compounds were used in six concentrations from 100 ppm to 3.125 ppm with 2-fold serial dilution. The same amount of DMSO was used as the negative control for all testing concentrations. Approximately 20 ± 2 individual *A. amphitrite* cyprids were placed into each well of the 24-well polystyrene culture plate containing 2 mL of FSW and were subjected to different treatments. For all treatments and controls, three replicates were performed. The plates were then incubated at 25 °C in darkness. After 48 h, the number of settled and swimming larvae were counted using a Leica MZ6 microscope, and possible toxic effects were noted.

### 3.6. Settlement Bioassay of H. elegans

The test compounds were dissolved with a small amount of DMSO. The test compounds were used at six different concentrations with a 2-fold serial dilution from 100 ppm to 3.125 ppm. The same amount of DMSO was used as the negative control for all testing concentrations. Approximately 10 ± 2 individual *H. elegans* larvae were placed into each well of a 24-well polystyrene culture plate that contained 2 mL of FSW with different concentrations of the test solution. Approximately 10^−4^ molarity of 3-isobutyl-l-methylxanthine was added into each well as an inducer for the settlement of *H. elegans* larvae [7]. The plates were then incubated at 25 °C in darkness. After 24 h, the number of settled and swimming larvae were counted using a Leica MZ6 microscope, and possible toxic effects were noted.

### 3.7. Determination of Working Concentration and Stability Using High-Performance Liquid Chromatography (HPLC)

The measurement and analysis of butenolide and Boc-butenolide were performed according to previous reports [13,21,22]. The preparation of nominal concentrations for butenolide and Boc-butenolide was described in the section of “Chemicals and seawater”. The calibration standards were prepared by serial dilutions of stock concentrations of butenolide and Boc-butenolide using methanol as the diluent, and the DMSO content in all calibration standard samples was lower than 0.5‰ *v*/*v*. The stock concentrations of butenolide and Boc-butenolide were made by dissolving 500 mg of butenolide or Boc-butenolide in 1 mL of DMSO to make a stock of 500 mg/mL and stored at −20 °C. The working concentrations of butenolide and Boc-butenolide samples used in settlement bioassay were measured by reverse-phase HPLC using a Waters 2695 separation module coupled to a Waters 2669 photo-diode array (PDA) detector according to the peak area at 210 nm (Waters Corporation, Taunton, MA, USA). Identification of butenolide and Boc-butenolide was determined based on their retention times (butenolide, 11 ± 0.1 min; Boc-butenolide, 6.8 ± 0.1 min). The samples were tested with a 20 min gradient of 50–99% aqueous acetonitrile (ACN) containing 0.05% *v*/*v* trifluoroacetic acid (TFA) at a flow of 1 mL/min. The working concentrations of butenolide and Boc-butenolide were calculated according to their standard curves using peak areas plotted against known quantities of standards. The recoveries for butenolide and Boc-butenolide were 90.9% and 99.5%, respectively.

The stability of Boc-butenolide was measured by the concentration changes of Boc-butenolide in ASW throughout 3 months. The starting nominal concentration of Boc-butenolide was 200 ppm. At every time point, 5 mL of the solution was drawn and mixed with 10 mL dichloromethane (DCM). The DCM fraction with the analyte was dried under nitrogen gas, redissolved reconcentrated in 1 mL of methanol and subjected to above HPLC analysis.

### 3.8. Preparation of Polymer/Antifoulant Coatings

The polymer/antifoulant coatings were prepared using the solution casting method described by Ding et al. [23]. The coating was prepared by dissolving poly(ε-caprolactone)-polyurethane (PCL-PU) [13] and butenolide or Boc-butenolide with different proportions (i.e., 95 wt% polymer and 5 wt% antifoulant for 5% antifoulant coating) in xylene, the mixture was then stirred vigorously until all solids dissolved, thereby forming a uniform solution. The coating solution was applied onto the surface of the panels, which were either epoxy panel (25 mm × 75 mm) for release rate determination or PVC panels (53 mm × 125 mm) for the field test. The panels were then placed under room temperature for 7 days until all solvents evaporated, and a continuous coating was formed.

### 3.9. Determination of Antifoulant Release Rate from the Coatings

The release rate of butenolide was determined by HPLC for quantification. The polymer/antifoulant coatings were prepared on an epoxy panel (25 mm × 75 mm) according to the above procedure. The coated panels were then placed into ASW. At certain time points (days 1, 8, 15, 22, 29, 50, 71 and 92 after immersion onto ASW), the panels were transferred into separate containers, which filled with 100 mL of fresh ASW. After 24 h immersion, the analyte in ASW was extracted with equal portion of dichloromethane (DCM). The DCM fraction with the analyte was dried under nitrogen gas, reconcentrated in 200 μL of methanol and subjected to HPLC analysis (Waters 2695, Taunton, MA, USA) using a reversed-phase system with a C18 column (Phenomenex Luna C18(2), 250 × 4.6 mm, 5 microns, Torrance, CA, USA) and a photodiode array detector (Waters 2998, Taunton, MA, USA) operated at 210 nm [13,21,22].

### 3.10. Field Test

The field tests were conducted in a fish farm in Yung Shue O, Hong Kong (114°21′ E, 22°24′ N) from January 2018 to March 2018. PVC panels (53 mm × 125 mm) covered with coatings were immersed in seawater at a depth of 1 m from the surface. The panels were retrieved once monthly, the dirt on the panels was removed by washing the panel gently with seawater before being photographed. The panels were placed back into the sea for monitoring. The antifouling potential of different panels were compared to determine the efficiency of the coatings. The estimation of the panel fouling coverage was achieved by ImageJ (National Institutes of Health, Bethesda, MD, USA) [24]. The percentage of area covered by foulers was calculated from the ratio of total fouling area to the panel area, in which the area was highlighted via the threshold function of ImageJ. IBM SPSS Statistics 22 was used for all statistical analyses. One-way ANOVA was used after initial analyses of heterogeneity and variance of the dataset with Levene’s test followed by Tukey’s post hoc test. Significance was defined as a *p*-value lower than 0.05.

## 4. Conclusions

The structure of butenolide was modified to Boc-butenolide to solve the problems of low melting point and smelliness of butenolide. *A. amphitrite* and *H. elagans* larval settlement bioassay results indicated that Boc-butenolide has similar antifouling ability against macrofoulers but with lower toxicity at high concentration. Boc-butenolide was released from the coatings and demonstrated antifouling ability for at least 2 months (as long as Boc-butenolide was released from coating). The release rate decreased with the increase in concentration of Boc-butenolide in the coatings. Our experiment demonstrated that Boc-butenolide exhibited good antifouling ability and could be a substitute compound for antifouling paints, and future efforts should focus on developing Boc-butenolide as a non-toxic antifouling compound with improved controlled release in the marine environment.

## Data Availability

The data presented in this study are available in the main text.
